# Does Tourism Induce Sustainable Human Capital Development in BRICS Through the Channel of Capital Formation and Financial Development? Evidence From Augmented ARDL With Structural Break and Fourier-TY Causality

**DOI:** 10.3389/fpsyg.2022.804349

**Published:** 2022-04-06

**Authors:** Jun Li, Md. Qamruzzaman

**Affiliations:** ^1^School of Economics and Business Administration, Chongqing University, Chongqing, China; ^2^School of Business and Economics, United International University, Dhaka, Bangladesh

**Keywords:** tourism, human capital development, financial development, gross capital formation AARDL, BRICS

## Abstract

The motivation of the study is to explore the nexus tourism-led sustainable human capital development (HCD) in Brazil, Russia, India, China, and South Africa (BRICS) for the period 1984–2019. The study applied several econometrical techniques for exposing the empirical association between tourism and HCD, such as the conventional and structural break unit root test, the combined cointegration test, long-run and short-run coefficients detected through implementing the Augmented Autoregressive Distributed Lagged (AARDL), and directional causality by following Toda-Yamamoto with Fourier function. The unit-roots test established variables are integrated in mixed order, wherein variables are stationary at a level or after the first difference. The estimated test statistics from the combined cointegration test and AARDL confirmed the long-run association between tourism, gross capital formation, financial development, and HCD. Tourism revealed a positive and statistically significant tie with HCD in the long run. Moreover, the joint effects of interactive terms TOR^*^GCF and TOR^*^FD (TOR, GCF, and FD denoting tourism development, gross capital formation, and financial development, respectively) established a positive and statistically significant relationship with HCD. In addition, the causality test revealed the feedback hypothesis available between tourism and HCD in all sample countries except India. In conclusion, the role of tourism development is critically important for sustainable HCD in BRICS. Therefore, in case of a policymaking concern, it is inevitable to address the tourism issues with care for capitalizing on the benefits for tourism development.

## Introduction

Economic development and poverty alleviation need a significant investment in human capital. This is because human capital accumulation boosts labor productivity, enables technological innovation, raises capital returns, and makes growth more sustainable, all of which contribute to poverty alleviation and ensure sustainable development (Gibescu, 2010). Thus, human capital accumulation is seen as a critical production element in the economy's overall production function at the macro level. From a microeconomic standpoint, education boosts one's employment seeking ability and increases their earning capability (Xia et al., 2021). Thus, human capital is defined at the micro-level as the component of education that adds to an individual's labor productivity and profits while also serving as a critical component of company output (Haltiwanger et al., 1999). In other words, human capital refers to people's capacity and efficiency in transforming raw resources and capital into products and services, and the agreement is that these talents may be acquired *via* education (Bhagavatula et al., 2010). Thus, human capital development (HCD) is critical for growth on its terms, not only for its instrumental usefulness.

Considering the existing literature focusing on HCD, it is apparent that two vines of evidence were available: the role of HCD in various macro aspects and the critical determinants of HCD in the economy. From the growth contributory perspective, human capital has been placed in the apex position among the other macro fundamentals for economic growth (Gebrehiwot, 2014; Matthew et al., 2018; Qamruzzaman et al., 2020), financial development (Nik et al., 2013; Khan et al., 2020), poverty alleviation (Becker, 1994; Adekoya, 2018), income inequality (Lee and Lee, 2018; Scheyvens et al., 2021), and environmental sustainability (Ma et al., 2017; Ahmad et al., 2021), among others. According to existing literature, HCD is one of the key determinants of economic resource optimization and sustainable development (Trostel et al., 2002), which is because human capital accumulation is attributed to economic growth. Over the past decades, researchers, academicians, and policymakers have invested time and money in exploring the micro-macro factors that have been playing a critical role in HCD, and they have also been able to derive a few factors such as level of education (Son, 2010), inequality (Londoño and Bank, 1996; Quang Dao, 2008), trade openness, foreign direct investment (FDI) (Ardichvili et al., 2012), financial development, remittances, institutional quality, and economic growth (Adelakun, 2011). It is apparent that the economic growth attributes have linkage with HCD, which is investigated in the literature. However, the nexus tourism-led HCD has yet to investigate in empirical studies extensively. Thus, the intended purpose of this study is to explore fresh insight concerning the nexus between tourism-led HCDs in selected Brazil, Russia, India, China, and South Africa (BRICS) countries. It is firmly believed that study findings enhance the conceptual understating of exploring the role of tourism development for human capital accumulation and open an avenue for the policy strategic rethinking process in tourism development. The novelty of the study is as follows:

First, tourism-led economic development (Muslija et al., 2017; Aratuo et al., 2019; Pan and Dossou, 2020), financial development (Ohlan, 2017), capital flows, FDI, and environment (Mishra et al., 2019; Dogru et al., 2020) have been extensively investigated, but the role on HCD still remains untouched. For the first time, the role of tourism receipts has been investigated for HCD in BRICS nations with our best knowledge. We firmly believe that the study findings will open an avenue for the strategic decision-making process in the case of tourism development and expedite the present state of HCD in BRICS.

Second, the role of gross capital formation has been investigated and we have assessed the impacts on various macro-fundamental. However, with our best knowledge for the first time, the direct impact of gross capital formation on HCD has been investigated with the study and has tried to establish a bridge for fulfilling the existing research gap.

Third, the study has incorporated the interactive terms for evaluating the indirect effects of tourism on HCD through the channel of gross capital accumulation and financial development. According to existing literature, tourism positively influences gross capital formation (Po and Huang, 2008; Hüseyni et al., 2017) and financial development (Kumar, 2014; Shahbaz et al., 2019), especially in the long run. The interactive term explained the joint effects on target variables. Therefore, the finding of interactive terms on HCD can augment the capital accumulation through skills development in the population.

According to the combined cointegration test and Augmented Autoregressive Distributed Lagged (AARDL) estimation, the findings of this study established a long-run association between tourism, gross capital formation, financial development, and HCD in sample countries' estimation. Referring to the long-run association, especially from tourism, gross capital formation, and financial development with HCD, the study documented positive and statistically significant links between explanatory variables that tourism, gross capital formation, and financial development have with HCD in both the long-run and short-run. Furthermore, directional causality with Fourier-Toda-Yamamoto (TY) revealed the feedback hypothesis that bidirectional causality runs between tourism and HCD [TOR← → HCD], suggesting the importance of both factors in their respective development.

The remaining structure of the manuscript is as follows: Section Literature Review deals with the literature review and conceptual development for the study. Data and econometrical tools are displayed in Section Methodology and Data of the Study. Empirical model estimation and interpretation are reported in Section Estimation and Interpretation. Discussion of the study findings are reported in Section Discussion and the conclusion is available in Section Conclusion.

## Literature Review

Since the advent of the growth model offered by Solow (1956), education has been seen as a significant driver of economic development. Even though education did not explicitly include in his development theory, the critical position of technology in his model gave momentum for the emphasis on education since a well-educated populace was required for technological innovation after all. Nelson and Phelps (1966) made the connection explicit in what they called “investment in humans,” wherein economy demands a skilled workforce for application and use of new technology, and education facilities enable the population to enrich their knowledge and skills, thereby boosting total factor productivity that drives economic expansion. According to new growth theories, such as those proposed by Lucas (1988), Romer (1990), and Mankiw et al. (1992), human capital accumulation through knowledge and skills development augment economic growth by improving labor productivity, promoting technological innovation and adaptation, and lowering fertility (Scheyvens et al., 2021).

Human capital is essential to economic development and poverty alleviation (Becker, 1995; Olopade et al., 2019). From a macroeconomic standpoint, human capital accumulation enhances economic productivity, enables technical breakthroughs, raises returns on capital, and makes growth more sustainable, which aids in poverty alleviation. Thus, human capital is a critical component of the macroeconomic production function. Micro-economically, education increases one's chances of finding work and increases earning potential. Thus, human capital is defined, at the microeconomic level, as the component of education that increases an individual's labor productivity and earnings while simultaneously serving as a vital component of business production. Human capital, in other words, refers to people's ability and efficiency in turning raw materials and capital into goods and services, and it is commonly believed that these qualities may be gained *via* the educational system. On the other hand, HCD is critical for development not just for its instrumental value but also for its intrinsic significance as a development target in and of itself.

Capital accumulation results in technological advancement in an economy, enhancing the advantages of large-scale manufacturing and increasing economic specialization (Ongo and Vukenkeng, 2014). Additionally, when capital development results in the efficient exploitation of natural resources and the construction of diverse businesses, income levels rise, allowing for the satisfaction of people's varied desires. As a result, it enhances residents' economic wellbeing and serves as a barometer of economic progress (Jena and Sethi, 2021). Furthermore, domestic capital accumulation enables a nation to achieve self-sufficiency and alleviates the weight of foreign debt. Inadequate domestic capital availability encourages nations to borrow money from another country for an extended period; it severely burdens future generations. As a result, the tax burden grows, and money leaves the economy through debt repayments. This indicates that only capital production results in independence from foreign help, a reduction in the weight of foreign debt, and self-sufficiency for the nation, which eventually accelerates the economy.

Capital formation has induced domestic aggregated output by promoting domestic trade liberalization, FDI inflows, and skills human resources development. Akobeng (2017) documented that gross capital formation in the economy assists in reducing the poverty level by allowing excess earning with grabbing investment opportunities, which eventually increase the speed of HCD. Gibescu (2010) advocated that gross capital formation plays a critical role in supplying the factors of production in the economy and in expediting economic growth toward sustainable development. The human element plays a role in economic development by increasing macroeconomic work volume and the quality of labor productivity, which is a synthetic expression of that work volume.

The study of Schumpeter (1911) opened the discussion on the role of financial markets in the economy with evidence that the finance-growth nexus is vital for economic sustainability. Several economists believe that financial markets are vital to economic growth because of the efficient financial intermediation, hence economic performance (Shaw, 1973; McKinnon, 1979; Levine, 1997), among others. Aziz and Duenwald (2002) outlined three ways financial development might affect economic growth. A well-developed financial market improves the efficiency of capital. Second, it enhances credit availability in the economy, and third, it lowers capital costs. It promotes effective financial intermediation between lenders and borrowers. Following then, an expanding body of literature began concentrating on people's living standards and wellbeing with the progress of financial development in the economy. Ranis (2004) found a link between HCD and rapid economic growth in his research. According to Sehrawat and Giri (2017), the financial sector and HCD are critical components of economic growth. The development of the financial industry in the absence of adequate human capital results in poor economic growth.

The studies conducted by De Gregorio (1996); Outreville (1999); Evans et al. (2002), and Papagni (2006) pioneered the study of the relationship between human capital and financial growth. Financial factors have a vital impact in boosting human capital in Pakistan. Broad money supplies have a significant impact on human development. However, a poor link between market capitalization and human capital was discovered. Similarly, Sethi et al. (2019) discovered that a higher rate of financial sector expansion and a big market size boost HCD in South Asia. Nik et al. (2013), on the other hand, investigated the link between human capital and financial growth in Iran. Because of the financial flows, they discovered an inverse link between both variables. The research investigated how inefficient banking channels, such as insufficient resource allocation, inadequate facilities, and so on, negatively impact human development.

Similarly, Hakeem and Oluitan (2012) discovered a detrimental impact of financial development on human capital in South Africa owing to their inefficient banking systems. On the other hand, some research found no substantial association between financial development and HCD (Hatemi and Shamsuddin, 2016). According to the existing nexus, human capital alongside financial growth revealed inconclusive evidence, suggesting the role of HCD in various economic structure, governmental practices, and institutional quality. HCD may help finance develop by closing knowledge gaps and rising demand for various financial products Hatemi and Shamsuddin (2016). Financial development is seen as vital as human capital contributing to economic progress. Arif and Khan (2019) have performed and studied for gauging the role of financial development on human capital accumulation in Pakistan for the period 1991–2016. Study findings documented that the financial institution's growth augmented HCD by releasing the effects of liquidity constraints and boosting technical skills development among the population. Another study conducted by Hakeem and Oluitan (2012) in South Africa study revealed that capital support with an efficient financial system allows both physical and HCD in the economy in the long run. Sehrawat and Giri (2017) underline that, when physical capital is paired with deficient human capital, economic development may be low. Only when finance is permitted to “do what finance can do” is physical capital anticipated to contribute to human capital. As a result, efficiency is boosted by redistributing buying power from low-return customers to high-return ones. Additionally, finance is anticipated to combat poverty by boosting income and, in the long term, through improving health and education (Yang et al., 2021). This is because investment in skill development and services and physical infrastructure that promote health and lifespan is critical.

### Conceptual and Hypotheses Model for Hypothesis Testing

**Figure 1:**
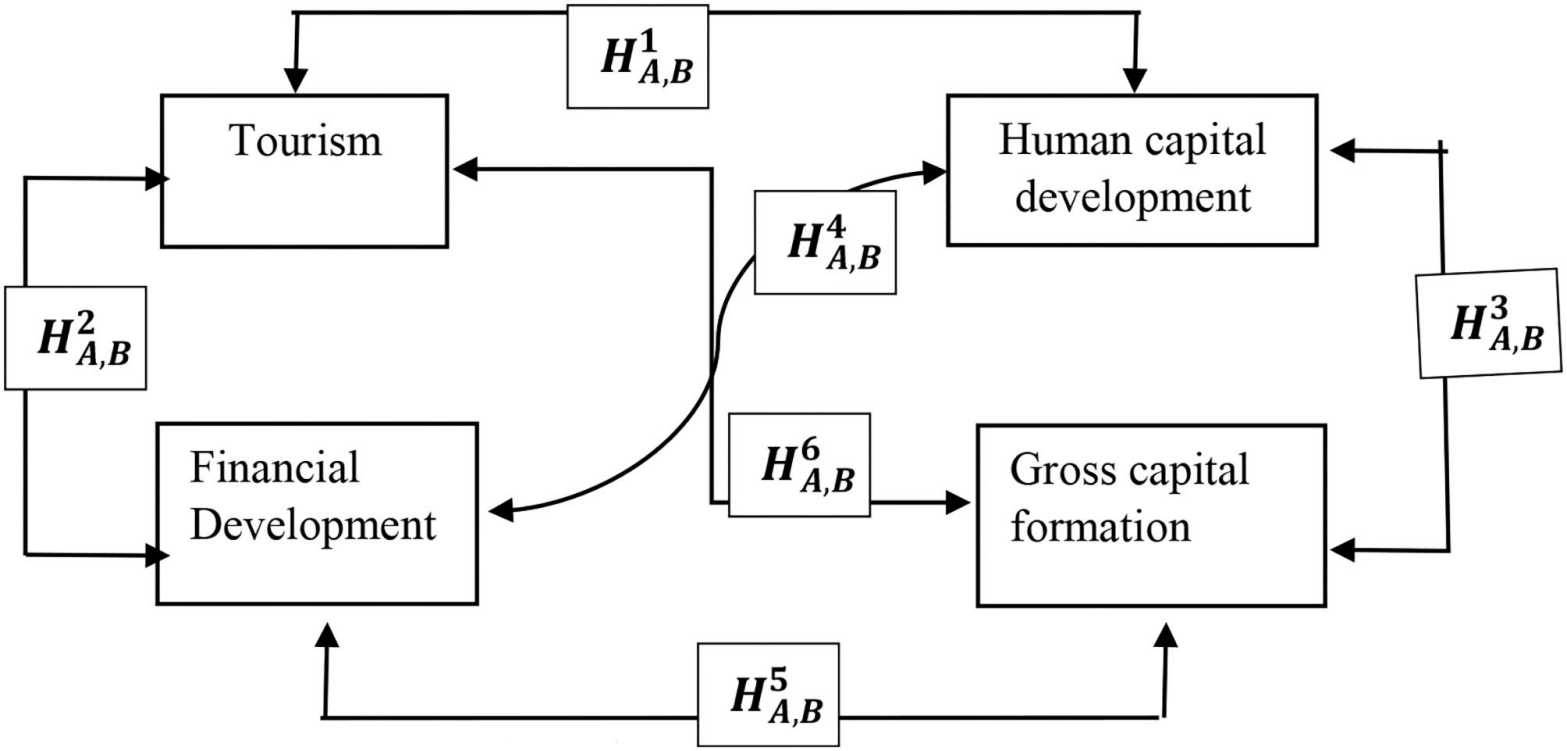
Conceptual model and hypothesis of the study.

Existing literature has revealed growing evidence, focusing on two-directional studies. First, a group of researchers have investigated the role of HCD on fundamental macro growth, such as economic growth, trade openness, remittances, financial development, and others. Second, similar to this study, assessing the key determinants of HCD in the economy and revealing several factors have been the focus. The motivation of the current study is to gauge the role of tourism development in the process of human capital accumulation in BRICS. It is evident in literature that skilled human resource availability promotes tourism across the world, but when the economy is talking about HCD, the role of tourism has yet to be revealed in extensive empirical investigation. Furthermore, this study also intended to look into the joint effects of tourism and capital formation and tourism and FDI on HCD. The motivation for joint effects was also studied when it comes to empirical estimation for exploring the indirect effects of gross capital formation and FDI since researchers have documented that tourism boosts the gross capital formation and FDI. Considering the study's motivation, we propose the following conceptual model (Figure 1) for understanding and hypothesis development.

The following hypothesis is to be tested in evaluating the directional causalities.

$H_{A,B}^{1}$: Tourism development Granger causes HCD and vice versa

$H_{A,B}^{2}$: Financial development Granger causes tourism development and vice versa

$H_{A,B}^{3}$: Gross capital formation Granger causes HCD and vice versa

$H_{A,B}^{4}$: Financial development Granger causes HCD and vice versa

$H_{A,B}^{5}$: Financial development Granger causes gross capital formation and vice versa

$H_{A,B}^{6}$: Tourism development Granger causes gross capital formation and vice versa.

## Methodology and Data of The Study

### Model Specification

The motivation of the study is to gauge the role of tourism on HCD in BRICS for the period 1980–2019 through the channel of capital formation and financial development. In investigating the nexus tourism-led HCD, BRICS has been chosen because tourism is an important area of cooperation among these countries. Cooperation in tourism increases people-to-people exchanges among the BRICS countries, leading to friendship fraternity and a better understanding of each other's culture and heritage. Furthermore, in recognizing the potential of tourism to contribute toward sustainable and socio-economic development, the 2013 BRICS eThekwini Declaration and Action Plan mentioned tourism as one of the new areas of cooperation to be explored by the BRICS countries. This was also reiterated in the Xiamen Declaration in 2017.

Taking into account the empirical nexus, the generalized model is as follows:


(1) $$\begin{array}{l} \text{HCD}|\text{ tourism},\text{ financial development},\text{ capital formation} \end{array}$$

(2) $$\begin{array}{l} \text{HCD|TOR},\text{FD},\text{GCF},{\text{TOR}}^{*}\text{FD},{\text{GCF}}^{*}\text{TOR} \end{array}$$

Equation (1) deals with the direct effects running from explanatory variables to dependent variables in the equation, but the interactive term incorporation in Equation (2) confirms to reveals both direct and indirect effects of tourism on HCD. The variables delimitation and data sources are displayed in Table 1.

**Table 1:** Variable definition and data sources.

**Variables**	**Notation**	**Definition**	**Sources**
Tourism	*Tor*	Tourism receipt as % of GDP	WDI
Human capital development	*HCD*	Human capital index, based on years of schooling and returns to education.	Penn World Table version 10.0., 2021
Capital formation	*GCF*	Gross capital formation as a % of GDP	WDI
Financial development	*FD*	Financial development index	IMF
Money supply	*MS*	Broad money as a % of GDP	WDI
Foreign Direct Investment	*FDI*	FDI inflows as a % of GDP	WDI

#### Variable Definitions and Descriptive Statistics

##### Human Capital Development

HCD is the process of enhancing human potential to achieve a healthy and knowledgeable life, a good quality of living, and the capacity to prosper (Becker and Gerhart, 1996). As previously said, HCD comprises the growth of human capability. The human development index (HDI) is a commonly used metric for assessing HCD. It is divided into three dimensions: education, health, and level of life.

##### Tourism

The term “tourism development” refers to a growth in the number of visitors visiting a nation over time. Increased tourist receipts are connected with tourism development. Tourism receipts are the sum of all expenditures made by visitors who visit a nation during a specific period:

##### Gross Capital Formation

Nwanna (1986) defined capital formation as the accumulation of both physical and intangible assets, such as plants, equipment, and machines, as well as intangible assets, such as high levels of education, health, and scientific knowledge. According to Kuznets (1955), domestic capital creation comprises additions to domestic structures, equipment, inventories, and additional capital expenditures. Capital accumulation is sometimes linked with earnings or savings, particularly tangible capital goods. Capital creation necessitates that a society or a nation devotes a portion of its existing economic activity to producing capital goods, such as tools and instruments, machinery and transportation infrastructure, and plant and equipment. In other words, it is the allocation of a portion of society's existing available resources to build the stock of capital goods to facilitate future increases in consumable output. Capital formation/accumulation is equivalent to investing in essence.

##### Financial Development

Financial development is an independent variable seen as a critical economic component. It is defined as the growth of the country's financial markets. A well-functioning financial system benefits the economy by enabling effective intermediation.

##### Foreign Direct Investment

The amount of FDI inflows is positively related to a country's economic development (Alfaro et al., 2004; Ajayi, 2006; Zhu et al., 2016; Ferdousi and Qamruzzaman, 2017). The need for skilled workers and qualified professionals to manage technical, managerial, and professional jobs has grown as FDI inflows have increased. The development of human capital is thus critical for sustainable social and economic development. FDI flows into emerging nations, and transition economies continue to grow at a breakneck pace. The primary reason for such nations receiving a disproportionate amount of FDI is their substantial investment in knowledge, which resulted in developing a trained labor force capable of embracing technical breakthroughs (Qamruzzaman et al., 2021). FDI is seen favorably by developing nations as a source of finance. Inadequate skills and training adversely affect FDI, reducing capital inflows to the host nation. Countries having a greater human capital pool attract a greater amount of FDI. The study of Gökmenoglu et al. (2018) advocated that FDI inflows accelerate the poverty allocation process by increasing the skilled workforce in the economy. Furthermore, Iamsiraroj and Ulubaşoglu (2015) documented that FDI inflows augmented HCD by increasing secondary school enrolment in the economy. In a nutshell, the literature suggested that FDI inflow has a positive effect on HCD across the world, which motivated us to incorporate FDI as a control variable in empirical estimation (Qamruzzaman et al., 2021).

##### Money Supply

In the empirical literature, the role of money supply in the economy has been investigated and documented, such as money supply led inflation, capital accumulation, both physical and human (Olanipekum and Akeju, 2013), economic growth (Doorasamy and Wilfred, 2020), and remittances (Kim, 2019). The study of Cover (1992) investigated the asymmetric effects of output level in the economy. The study documented that positive shocks in money supply increase output level, whereas adverse effects revealed negative shocks in the money supply. In the study of Ezeaku et al. (2018), it was postulated that money supply plays a deterministic role in industrial output acceleration through direct and indirect channels, that is, the optimal level of money supply control inflation and accelerate domestic trade expansion of financial development.

The motivation of the study is to investigate the role of tourism and gross capital formation on HCD in BRICS for the period 1980–2019. By taking into account the variables mentioned above, the following generalized Equation (1) is to be implemented to explore each variable's elasticity


(3) $$\begin{array}{l} HCD_{t}=\alpha_{0}+\beta_{1}TOR_{t}+\beta_{2}GCF_{t}+\beta_{3}FD_{t}+{\beta_{4}X}_{t}^{{\;}^{*}}\varepsilon_{t}\ldots , \end{array}$$

with interactive term for joint effects


(4) $$\begin{array}{l} HCD_{t}=\alpha_{0}+\gamma_{1}TOR_{t}+\gamma_{2}GCF_{t}+\gamma_{3}FD_{t}+\gamma_{4}{\left(FD_{t}*TOR\right)}_{t}\\ \;+\gamma_{5}{\left(GCF_{t}*TOR\right)}_{t}+\gamma_{6}X_{t}^{*}\varepsilon_{t}+\;\varepsilon_{t}\ldots , \end{array}$$

where HCD stands for human capital development, TOR for tourism development, GCF denotes gross capital formation, FD for financial developmen (FD^*^TOR) and (TOR^*^GCF) are interactive terms, and X denotes control variables that are FDI and money supply in the economy. E is the error term, and subscript *t* is the period. All the data were transformed into a natural log before empirical estimation.

#### Estimation Strategy

##### Unit Root Test

In empirical model estimation considering time series data, the properties of the variable assessments are critically essential for selecting the appropriate strategies for evaluating the nexus between dependent and explanatory variables (Meng et al., 2021). The study applied several unit root tests for evaluating the variable's stationarity properties, such as the Augmented Dickey-Fuller (ADF) test (Dickey and Fuller, 1979), the Philipps-Perron (P-P) test (Phillips and Perron, 1988), the Dickey-Fuller Generalized Least Square (DF-GLS) test (Elliott et al., 1996), the Kwiatkowski–Phillips–Schmidt–Shin (KPSS) test (Kwiatkowski et al., 1992), and the Zivor-Andrews (Z-A) test (Zivot and Andrews, 2002) for one structural break in the research unit.

The ADF test has investigated the stationary properties with a lagged difference form of target variable so that serial correlation can be addressed. The following system function is to be considered.


(5) $$\begin{array}{l} \Delta Y=\gamma_{0}+\gamma_{1}Y_{t-1}+{y2}^{t}+\overset{w}{\underset{i=1}{\sum }}\alpha_{i}{\Delta Y}_{t-1}+\mu_{t}, \end{array}$$

Elliott et al. (1996) extended the ADF test and used the DF-GLS test, performed based on ordinary least square (OLS). The stationary test through DF-GLS allows linear trend in assessment, which is as follows:


(6) $$\begin{array}{l} {\Delta y}_{t}^{d}=\alpha y_{t-1}^{d}+\overset{p}{\underset{i=1}{\sum }}\vartheta_{j}{\Delta y}_{t-i}^{d}+\sigma_{t}, \end{array}$$

where $y_{t}^{d}$ stands for de-trend data and σ_*t*_ stands for the white noise error term.

Kwiatkowski et al. (1992) familiarized the unit root test with the null hypothesis of stationary by implementing the following time series mode:


(7) $$\begin{array}{l} y_{t}=\beta_{0}+\beta_{1}t+\gamma_{t}+\epsilon_{t} \end{array}$$

(8) $$\begin{array}{l} \gamma_{t}=\gamma_{t-1}+\theta_{t}, \end{array}$$

*where β*_0_ and β_1_ explain the deterministic term in a constant form and a linear trend in Equation (7), whereas γ_*t*_ stands for the random walk factors in the estimation. Kwiatkowski et al. (1992) proposed the following Lagrange multiplier (LM) test statistics for stationary tests.


$$\begin{array}{l} LM=\frac{1}{T^{2}}\frac{\sum_{t-\;1}^{T}M_{t}^{2}}{\hat{\delta^{2}}}, \end{array}$$

where $M_{T}=\overset{t}{\underset{i=1}{\sum }}e_{i}$ stands for residuals from OLS estimation and δ^2^ is the variance estimator, which may remove nuisance parameters from the asymptotic distribution of the LM statistics under the null hypothesis.

#### Bayer and Hacked Combined Cointegration Test

In investigating the long-run association in empirical assessment, the conventional cointegration was extensively used prior to the inception of the novel combined cointegration test. In some instances, the conclusion reached with different cointegration tests revealed inconclusive decisions. Therefore, the so-called Bayer-Hanck test was newly proposed by Bayer and Hanck (2013) by ensuring the power of cointegration test, with the unique aspect of generating a joint test-statistic for the null hypothesis of no cointegration based on Engle and Granger, Johansen, Peter Boswijk, and Banerjee tests. Since this new approach allows us to combine various individual cointegration test results to provide a more conclusive finding, it is also applied in this study to check the presence of a cointegrating relationship between tourism, gross capital formation, foreign direct investment, financial development, money supply, and HCD in BRICS nations.

The study implemented the cointegration test by following the framework proposed by Bayer and Hanck (2013), commonly known as the combined cointegration test. The proposed cointegration test consists of four conventional tests of cointegration familiarized by Engle and Granger (1987), Johansen (1991), Peter Boswijk (1994), and Banerjee et al. (1998), and with the null hypothesis of the no cointegration test, and the following Fishers' equation is considered in deriving the test statistics for detecting long-run association.


$$\begin{array}{l} EG-JOH=-2[LN\left(PEG\right)+LN\left(PJOH\right)]\\ EG-JOH-BO-BD=-2[LN\left(PEG\right)-\ln\left(PJPH\right)\\ \;+\ln\left(PBO\right)+\ln\left(PBDM\;\right)], \end{array}$$

where PBDM, PBO, PJOH, and PEG stand for the significance levels of Engle and Granger (1987), Johansen (1991); Boswijk (1995), and Banerjee et al. (1998), respectively.

### Augmented ARDL

In recent times, by investigating long-run association in empirical studies, the framework proposed by Pesaran known as ARDL was extensively applied to see (Qamruzzaman and Jianguo, 2018; Qamruzzaman et al., 2020; Qamruzzaman and Karim, 2020a,b). ARDL estimation possesses certain benefits over traditional cointegration tests, those are, (1) efficient estimation regardless of the study's sample size (Ghatak and Siddiki, 2001), (2) capability of handling mixed-order variable integration, and selecting appropriate lagged specifications for model stability and efficiency (Pesaran et al., 2001), and (3) unbiased estimation for both long-run and short-run elasticity (Banerjee et al., 1993) (see Table 2).

**Table 2:** The definition of null hypotheses for all three tests.

**Cointegration test**	**Null hypothesis**	**Alternative hypothesis**
F-bound test	γ_1_ = γ_2_ = γ_3_ = γ_4_ = 0	Any, *u*_1_, *u*_2_, *u*_3_, *u*_4_ ≠ 0
A *t*-test on lagged dependent variable	γ_1_ = 0	γ_1_ ≠ 0
F-test on the lagged independent variable	γ_2_ = γ_3_ = γ_4_ = 0	Any, break *u*_2_, *u*_3_, *u*_4_ ≠ 0

Following Pesaran et al. (2001), the Equations (3) and (4) transformed into a generalized ADRL model for investigating the role of tourism on HCD through the channel of capital formation and financial development.


(9) $$\begin{array}{l} {\Delta lnHCD}_{t}=\alpha_{0}+\overset{n}{\underset{i=1}{\sum }}\mu_{1}{\Delta lnHCD}_{t-i}+\;\overset{n}{\underset{i=0}{\sum }}\mu_{2}{\Delta lnTOR}_{t-i}\\ +\overset{n}{\underset{i=0}{\sum }}\mu_{3}\Delta lnCF_{t-i}+\overset{n}{\underset{i=0}{\sum }}\mu_{4}\Delta lnFD_{t}\\ +\overset{n}{\underset{i=0}{\sum }}\mu_{5}\Delta lnGCF_{t-i}+\overset{n}{\underset{i=0}{\sum }}\mu_{6}\Delta lnX_{t-i}\\ +\gamma_{1}lnTOR_{t-1}+\gamma_{2}lnCF_{t-1}\\ +\gamma_{3}lnFD_{t-1}+\gamma_{4}lnX_{t-1}+\omega_{1t} \end{array}$$

(10) $$\begin{array}{l} {\Delta lnHCD}_{t}=\alpha_{0}+\overset{n}{\underset{i=1}{\sum }}\mu_{1}{\Delta lnHCD}_{t-i}+\;\overset{n}{\underset{i=0}{\sum }}\mu_{2}{\Delta lnTOR}_{t-i}\\ +\overset{n}{\underset{i=0}{\sum }}\mu_{3}\Delta lnCF_{t-i}+\overset{n}{\underset{i=0}{\sum }}\mu_{6}\Delta lnFD_{t}\\ +\overset{n}{\underset{i=0}{\sum }}\mu_{7}\Delta ln{CF\;*\;TOR}_{t-i}+\overset{n}{\underset{i=0}{\sum }}\mu_{7}\Delta ln{FD\;*\;TOR}_{t-i}\\ +\gamma_{1}lnTOR_{t-1}+\gamma_{2}lnCF_{t-1}+\gamma_{3}lnFD_{t-1}\\ +\gamma_{4}{lnFD\;*\;TOR}_{t-1}+\gamma_{5}{lnCF\;*\;TOR}_{t-1}+\omega_{1t} \end{array}$$

where Δ indicates differencing of variables, while is the error term (white noise), and (t-1) is for the lagged period. Based on linear ARDL, the long-run coefficient were available from γ_1_ to γ_5_ and short-run coefficients were obtained from μ_1_to μ_5_ from each empirical model estimation. Long-run association between variables were tested following the F-test (Pesaran et al., 2001) and the lagged level of the dependent variable following the *t*-test, as suggested by and the lagged levels of the independent variable(s) following another additional F-test as suggested by McNown et al. (2018).

The study implemented the following equation with error correction terms to capture the short-run dynamics.


(11) $$\begin{array}{l} {\Delta lnHCD}_{t}=\alpha_{2}+\overset{n}{\underset{i=1}{\sum }}\beta_{1}{\Delta lnHCD}_{t-i}+\;\overset{n}{\underset{i=0}{\sum }}\beta_{2}{\Delta lnTOR}_{t-i}\\ +\overset{n}{\underset{i=0}{\sum }}\beta_{3}\Delta lnR_{t-i}+\overset{n}{\underset{i=0}{\sum }}\beta_{6}\Delta lnFDI_{t}\\ +\overset{n}{\underset{i=0}{\sum }}\beta_{7}\Delta lnGCF_{t-i}+\rho ECT_{t-1}+\omega_{1t} \end{array}$$

(12) $$\begin{array}{l} {\Delta lnHCD}_{t}=\alpha_{2}+\overset{n}{\underset{i=1}{\sum }}\beta_{1}{\Delta lnHCD}_{t-i}+\;\overset{n}{\underset{i=0}{\sum }}\beta_{2}{\Delta lnTOR}_{t-i}\\ +\overset{n}{\underset{i=0}{\sum }}\beta_{3}\Delta lnGCF_{t-i}+\overset{n}{\underset{i=0}{\sum }}\beta_{4}\Delta lnFD_{t}\\ +\overset{n}{\underset{i=0}{\sum }}\beta_{5}\Delta lnX_{t-i}+\overset{n}{\underset{i=0}{\sum }}\beta_{6}\Delta ln{TOR\;*\;GCF}_{t-i}\\ +\overset{n}{\underset{i=0}{\sum }}\beta_{7}\Delta ln{TOR\;*\;FD}_{t-i}+\rho ECT_{t-1}+\omega_{1t} \end{array}$$

Researchers used the Granger (1969) causality test to look into causal relationships between macroeconomic variables. However, structural discontinuities in the series are ignored by the Granger test and many other causality tests in the literature, including those by TY (Toda and Yamamoto, 1995). Enders and Jones (2016) demonstrated that incapacity to account for structural breaks leads to misspecification issues in the vector autoregression (VAR) model. As a result, deviations toward the erroneous rejection of the true null hypothesis arise. The Fourier-TY causality tests were developed by Nazlioglu et al. (2016) to compensate for this omission with the extension of the trigonometric term, and the VAR model can be reproduced in the following ways:


(13) $$\begin{array}{l} y_{t}=\alpha \left(t\right)+\beta_{1}y_{t-1}+\ldots +\beta_{p+d}y_{t-\left(p+d\right)}+\varepsilon_{t} \end{array}$$

where α(*t*) explain the possible structural changes in the dependent variable (y), β_1_ stands for the coefficients, and ε_*t*_ stands for the white noise error term in the equation. The above Equation (13) can be transformed with Fourier functions for capturing the unknown structural changes in the following manner.


(14) $$\begin{array}{l} y_{t}=\alpha \left(t\right)+\beta_{1}y_{t-1}+\ldots +\beta_{p+d}y_{t-\left(p+d\right)}\\ +\vartheta_{1}\sin\frac{2k\pi t}{T}\;+\vartheta_{2}\cos\frac{2k\pi t}{T}\;+\varepsilon_{t} \end{array}$$

where k refers to the frequency, t denotes time trend, T shows the number of observations, and %_ and %_ measures the amplitude and displacement of the frequency. The null hypothesis for Fourier–TY test is no causality between variables (*H*_0_ : β_1_ = β_2_…………….β_*P*_ = 0).

## Estimation and Interpretation

### Unit Root Test

Detection of variable properties is one of the critical strategic decisions for appropriate selection of econometrical model; thus, we begin by implementing the test of stationery, the unit root test. The study implemented both conventional unit root test following Dickey and Fuller (1979), Phillips and Perron (1988), unit root following Kwiatkowski et al. (1992) and Elliott et al. (1996), as well as unit root test following Ng and Perron (2001) and unknown structural break unit root test following (Zivot and Andrews, 2002). The conventional unit root test displayed in Table 3 reveals that few variables are stationary at a level, but all the variables become stationary after the first difference; it was suggested that few variables can be used directly into the empirical estimation without derivation, but according to stationary test, some variables need to be derived with differential for empirical assessment. However, neither variables were exposed to stationary after the second difference. The conclusion is valid for all counties in the study samples.

**Table 3:** Results of conventional unit root test.

	**At level**				**After first difference**			
	**ADF**	**GF-DLS**	**PP**	**KPSS**	**ADF**	**GF-DLS**	**PP**	**KPSS**
Panel-A: for brazil								
HCD	−0.461	−1.757	−0.027	0.9280	−7.346	−4.181	−4.199	0.1570
TOR	−1.671	−0.239	−1.871	0.7600	−4.554	−3.588	−4.297	0.0770
CF	−0.908	−1.717	−0.165	0.9510	−6.919	−2.502	−4.053	0.1240
FD	−1.287	−0.85	−1.314	0.8070	−5.764	−2.394	−4.166	0.1310
MS	−2.145	−1.821	−1.538	0.8600	−5.077	−2.385	−4.535	0.1060
FDI	−2.337	−1.455	−0.505	0.7060	−4.019	−4.896	−4.769	0.1510
Panel-B: for Russia								
HCD	−1.866	−1.468	−2.714	0.7950	−7.98	−3.915	−3.235	0.1840
TOR	−2.617	−2.217	−1.319	0.6890	−4.666	−3.696	−5.123	0.1810
CF	−1.121	−1.237	−0.249	0.8610	−6.973	−4.532	−3.525	0.1120
FD	−0.32	−0.325	−2.505	0.8360	−5.074	−4.495	−4.467	0.0960
MS	−1.201	−1.063	−2.496	0.7780	−7.85	−3.277	−5.348	0.1450
FDI	−0.347	−2.429	−1.753	0.7170	−7.243	−2.133	−3.103	0.0990
Panel-C: India								
HCD	−2.751	−2.119	−0.215	0.7700	−4.591	−2.996	−5.876	0.0990
TOR	−0.152	−2.776	−1.73	0.9180	−4.544	−2.748	−3.783	0.1610
CF	−1.82	−0.451	−1.958	0.9070	−4.841	−3.193	−3.411	0.1310
FD	−1.003	−2.121	−2.887	0.9380	−6.69	−4.619	−3.614	0.1360
MS	−1.545	−0.56	−2.867	0.7820	−6.879	−2.05	−3.248	0.0750
FDI	−1.138	−2.943	−1.323	0.7730	−4.669	−2.166	−5.629	0.1280
Panel-D: for China								
HCD	−2.15	−1.304	−2.235	0.7090	−6.079	−4.955	−3.516	0.1000
TOR	−2.35	−1.38	−2.965	0.8930	−5.204	−2.945	−4.732	0.1210
CF	−0.318	−0.428	−2.542	0.9070	−5.653	−4.251	−5.231	0.1470
FD	−0.463	−1.715	−1.42	0.9800	−5.124	−2.184	−5.295	0.1530
MS	−2.582	−2.629	−1.339	0.7490	−5.027	−2.08	−5.246	0.0890
FDI	−0.161	−1.336	−2.311	0.7820	−7.891	−3.36	−5.498	0.1030
Panel-E: for South Arica								
HCD	−0.477	−1.232	−1.033	0.8830	−5.815	−3.893	−4.063	0.0890
TOR	−1.841	−2.83	−1.656	0.7290	−4.854	−3.71	−5.595	0.1430
CF	−1.122	−0.031	−0.57	0.9690	−4.781	−3.296	−5.696	0.1590
FD	−0.629	−1.799	−0.164	0.7930	−6.916	−2.411	−3.947	0.1710
MS	−2.974	−1.474	−0.98	0.9700	−6.856	−2.435	−3.816	0.1080
FDI	−2.915	−0.487	−0.311	0.7990	−6.492	−4.544	−3.606	0.1660

The unit root test results with an unknown structural break are displayed in Table 4. According to test statistics, all the variables are stationary after first difference with one structural, particularly HCD, exposed stationary each with break year (optimal lag) for Brazil 2001(2), Russia 2014(1), India 2008(1), China 1999(1), and South Africa 2009(1).

**Table 4:** Results of unit root test with an unknown structural break.

	**Test statistics**	**Break point**	**lag**		**Test statistics**	**Break point**	**lag**
Panel-A: for Brazil							
HCD	−2.379	2015	1	ΔHCD	−5.044	2001	2
TOR	−2.55	2011	2	ΔTOR	−6.048	2010	1
CF	−2.713	2000	1	ΔCF	−5.086	2004	2
FD	−3.107	2013	1	ΔFD	−8.174	1997	3
MS	−2.328	2015	3	ΔMS	−5.551	2007	2
FDI	−1.902	2011	2	ΔFDI	−6.735	2000	2
Panel-B: for Russia							
HCD	−3.151	1998	2	ΔHCD	−5.698	2014	1
cTOR	−2.36	2011	2	ΔTOR	−8.78	2002	2
CF	−1.96	2005	2	ΔCF	−7.621	1998	2
FD	−2.944	2008	2	ΔFD	−7.581	2005	2
MS	−2.334	2014	3	ΔMS	−8.029	2016	3
FDI	−2.227	2002	3	ΔFDI	−8.155	1999	3
Panel-C: for India							
HCD	−2.805	1999	3	ΔHCD	−8.776	2008	1
TOR	−2.688	2007	3	ΔTOR	−8.598	2017	3
CF	−2.544	2012	3	ΔCF	−5.841	2012	1
FD	−2.473	2002	2	ΔFD	−7.578	2018	2
MS	−2.797	2010	3	ΔMS	−7.696	2006	2
FDI	−2.747	2010	3	ΔFDI	−7.506	1998	1
Panel-D: China							
HCD	−2.171	2003	2	ΔHCD	−7.783	1999	1
TOR	−2.627	2009	3	ΔTOR	−6.896	2014	1
CF	−2.752	2013	2	ΔCF	−7.868	2008	2
FD	−2.9	2005	2	ΔFD	−7.403	1998	3
MS	−2.436	2016	2	ΔMS	−7.038	1998	3
F	−2.813	2005	3	ΔFDI	−9.098	2002	1
Panel-E: for South Africa							
HCD	−2.291	2005	1	ΔHCD	−6.531	2009	1
TOR	−2.89	2000	2	ΔTOR	−6.303	2017	1
CF	−2.716	2012	3	ΔCF	−6.968	2002	3
FD	−3.01	2014	1	ΔFD	−8.695	2005	3
MS	−2.093	2018	3	ΔMS	−7.917	2010	2
FDI	−2.792	2009	1	ΔFDI	−6.794	2007	3

The following study moved to detect the long-run association in the empirical equation before implementing the target model by implementing the novel test of cointegration familiarized by Bayer and Hanck (2013). The result of the cointegration test is displayed in Table 5. It was found that all the test statistics of model [1] to [5] were higher than the offered critical value at a 5% level of significance, suggesting the long-run association available between HCD, TOR, GCF, FD, MS, and FDI in BRICS nations. Considering the novel cointegration test results, it is postulated that all the selected independent variables are critically important in ensuring sustainable HCD because the long-run association explained co-effects running between dependent and independent variables. Thus, any variations in any variables can be caused in either manner.

**Table 5:** Results of Bayer–Hacked combined counteraction test.

**Model**		**EG-JOH**	**EG-JOH-BO-BDM**
HCD|TOR		10.996	23.42
HCD|TOR	Brazil	10.996	23.42
	Russia	11.554	24.129
	India	13.125	27.341
	China	10.897	24.209
	South Africa	13.609	23.682
HCD|TOR, GCF	Brazil	11.021	27.541
	Russia	11.101	23.904
	India	12.31	25.94
	China	12.319	24.313
	South Africa	13.56	27.121
HCD|TOR, GCF, FD	Brazil	13.089	26.482
	Russia	14.109	24.61
	India	11.782	23.629
	China	11.587	27.275
	South Africa	13.405	25.047
HCD|TOR, GCF, FD, MB	Brazil	13.301	26.958
	Russia	10.892	23.127
	India	11.997	23.802
	China	13.299	26.436
	South Africa	12.321	26.269
HCD|TOR, GCF, FD, MB, FDI	Brazil	11.047	26.144
	Russia	12.718	24.879
	India	13.437	22.568
	China	12.91	27.272
	South Africa	14.239	23.831

### Empirical Model Estimation With Equation (1)

Next, we moved to gauge the long-run association between remittances, financial development, cash flows, and HCD by performing the Equation (9). The long-run association under the augmented ARDL framework is displayed in Table 6. The study documented that the test statistics of F_overall_, t_DV_, and F_IDV_ are statistically significant at a 1% significance, suggesting the long-run cointegration between research units. The conclusion of long-run association in the empirical model is valid for all sample countries' estimations. Once the cointegration has been detected, the study evaluates the long-run and short-run magnfititutes of explanatory variables on HCD in BRICS nations.

**Table 6:** Augmented Autoregressive Distributed Lagged (AARDL) cointegration test.

**Empirical model**		**Test statistics**	**Brazil**	**Russia**	**India**	**China**	**South Africa**
HCD|*TOR, FD, GCF, MS, FDI*		F*_*overall*_*	10.229^***^	9.847^***^	9.818^***^	8.811^***^	9.848^***^
		t_DV_	−7.771^***^	−7.83^***^	−8.117^***^	−8.207^***^	−6.586^***^
		F*_*IDV*_*	4.254^***^	4.883^***^	6.57^***^	4.321^***^	5.961^***^
*Critical value : K = 5*	1%		5%		10%		
	I(0)	I(1)	I(0)	I(1)	I(0)	I(1)	
Pesaran et al. (2001)	5.095	6.77	3.673	5.002	3.087	4.277	
Narayan (2005)	−3.96	−5.13	−3.41	−4.52	−3.13	−4.21	
Sam et al. (2019)	3.58	5.91	2.46	4.18	2.00	3.47	

*** *denote 1% level of significant*.

The empirical estimation results of the long-run and short-run coefficients are displayed in Table 7, with Panel A for long-run coefficients, panel B for short-run coefficients, and the residual diagnostic test for panel C. Referring to tourism effects on HCD, the study documented positive and statistically significant association in Brazil (a coefficient of 0.1728), in Russia (a coefficient of 0.1257), in India (a coefficient of 0.1713), in China (a coefficient of 0.1903), and in South Africa (a coefficient of 0.1751%). More precisely, a 10% growth in tourism development in terms of tourism receipts can result in augmenting the progress of the human capital accumulation process in BRICS nations by 1.728% in Brazil, 1.257% in Russia, 1.711% in India, 1.903% in China, and 1.751% in South Africa. The study findings suggest that continual inflows of tourism income in the economy can boost the speed of HCD and support sustainable economic growth. Our findings align with existing literature such as Ngoma and Ismail (2013). The short-run assessment has revealed a similar association line to the long-run assessment, that is positive and statistically significant. In the short run, a 10% growth in remittance receipts increases HCD by 1.02% in Brazil, by 0.93% in Russia, by 0.9365% in India, by 0.88% in China, and by 0.48% in South Africa. However, the short-run elasticities are less prominent compared with the long-run horizon.

**Table 7:** Results of long-run and short-coefficient with AARDL.

	**Brazil**	**Russia**	**India**	**China**	**South Africa**
Panel-A: Long-run coefficients					
TOR	0.1728 (0.0461) [3.7487]	0.1257 (0.0832) [1.5113]	0.1713 (0.0536) [3.1967]	0.1903 (0.0826) [2.3041]	0.1751 (0.018) [9.6871]
CF	0.0911 (0.057) [1.5955]	0.0979 (0.0231) [−4.1201]	0.1457 (0.0391) [3.7209]	0.1109 (0.0398) [2.7836]	0.1556 (0.0742) [2.0959]
FDB	0.1378 (0.0526) [2.6194]	0.0960 (0.3946) [2.4332]	0.1427 (0.096) [1.4855]	0.1681 (0.037) [4.5395]	0.0913 (0.0643) [1.4203]
FDI	0.248 (0.0117) [21.1156]	0.012 (0.0108) [1.1109]	0.1158 (0.0455) [2.5459]	0.0559 (0.0061) [9.0641]	−0.1814 (0.0594) [−3.0516]
MS	0.1305 (0.0847) [1.5402]	0.0751 (0.1653) [0.4541]	−0.0884 (0.0526) [−1.6813]	0.107 (0.0563) [1.8989]	0.1283 (0.0943) [1.3605]
DMU	−0.0742 (0.0368) [−2.0166]	−0.0088 (0.0066) [−1.3346]	0.0151 (0.0096) [1.5754]	0.2024 (0.0744) [2.7203]	−0.1673 (0.0443) [−3.7726]
C	2.1897 (0.9027) [2.4254]	2.638 (1.3638) [1.9343]	−0.4741 (0.091) [−5.2063]	−0.0974 (0.0203) [−4.7809]	−0.134 (0.0846) [−1.584]
Panel-B: Short-run coefficients					
TOR	0.102 (0.039) [2.6096]	0.093 (0.041) [2.2496]	0.0936 (0.0139) [6.7009]	0.088 (0.0494) [1.7806]	0.048 (0.0168) [2.8514]
CF	0.0153 (0.0037) [4.1717]	−0.0683 (0.0109) [−6.2523]	0.0104 (0.0012) [8.2747]	0.0125 (0.0055) [2.2558]	0.0129 (0.0019) [6.4671]
FD	0.0233 (0.0031) [3.7173]	0.0162 (0.0386) [0.4201]	0.058 (0.0091) [5.9959]	0.097 (0.0502) [1.9329]	0.0288 (0.0027) [10.3156]
FDI	0.0029 (0.001) [2.6839]	−0.0388 (0.0445) [−0.8708]	−0.0023 (0.0008) [−2.9291]	0.0792 (0.0085) [9.2646]	0.0144 (0.0018) [7.6939]
MS	0.0023 (0.0011) [2.141]	−0.0338 (0.001) [−32.3444]	−0.0079 (0.001) [−7.3314]	0.013 (0.0049) [2.6585]	−0.0059 (0.0013) [−4.5807]
DMU	−0.0033 (0.0012) [−2.7379]	0.0168 (0.0029) [5.7899]	−0.0824 (0.0055) [−14.914]	0.145 (0.0098) [14.8047]	−0.0841 (0.0068) [−12.2037]
CointEq (−1)*	−0.144 (0.003) [−44.4307]	−0.1212 (0.0045) [−26.8451]	−0.1584 (0.0102) [−15.5056]	−0.362 (0.0219) [−16.487]	−0.1164 (0.0109) [−10.6784]
Panel-C: Residual diagnostic test					
	0.5107	0.6826	0.528	0.4888	0.7222
	0.5492	0.5895	0.6382	0.412	0.4782
	0.6035	0.7945	0.4266	0.5925	0.5337
	0.5447	0.7586	0.4241	0.7635	0.4235

The role of capital adequacy in capital formation in the economy boosts the present state of human capital accumulation, suggesting the positive and statistically significant association between capital formation and HCD in BRICS nations. In particular, a 10% growth in domestic capital formation in BRICS can increase the process of human capital accumulation by 0.911% in Brazil, 0.979% in Russia, 1.457% in India, 1.109% in China, and 1.556% in South Africa. While referring to short-run coefficients, the positive effects are from gross capital formation to HCD. However, the magnfititutes are more evident in the long-run compared with the short-run. Precisely, a 10% innovation in gross capital formation in the short-run accelerates the HCD process by 0.153% in Brazil, 0.104% in India, 0.125% in China, and 0.129% in South Africa, whereas negative linkage was revealed in Russia with a coefficient of −0.683%.

For nexus between financial development and HCD, the study established a positive and statistically significant association in the long run (short run) in Brazil with a coefficient of 0.1378 (0.0233), in Russia with a coefficient of 0.096 (0.0162), in India with a coefficient of 0.1427 (0.0058), in China with a coefficient of 0.1681 (0.097), and in South Africa with a coefficient of 0.0913 (0.0288). Long-run magnfititutes of financial development are evident in comparison to the short-run assessment. More precisely, a 10% further development in the financial system can accelerate the growth of human capital accumulation in the economy by 1.378% in Brazil, 0.96% in Russia, 1.427% in India, 1.681% in China, and 0.913% in South Africa.

The coefficient of error correction established negative and statistically significant at a 1% level of significance, suggesting that long-run convergence toward the equilibrium position. More precisely, due to explanatory variables shock in the short-run, the disequilibrium state can be rectified at a speed of 14.4% per period in Brazil, 12.12% per period in Russia, 15.84% per period in India, 36.2% per period in China, and 11.64% per period in South Africa. Furthermore, the study has implemented several residual diagnostic tests for confirming the model's internal consistency, robustness in estimation, and efficiency in managing residual (see Panel-C). Referring to residual diagnostic test statistics, it was revealed that empirical models are free from serial correlation, and normally distributed residuals have no issue for heteroskadacity.

Next, we gauge the long-run cointegration between remittances, financial development, cash flows, and HCD by performing Equation (10) with the interactive term: TOR^*^FDD, TOR^*^GCF for indirect effects of tourism through the channel of financial development and capital formation in the economy. The long-run association under the augmented ARDL framework is dispalyed in Table 8. The study documented that the test statistics of F_overall_, t_DV_, and F_IDV_ are statistically significant at a 1% significance, suggesting the long-run cointegration between research units. The conclusion of long-run association in the empirical model is valid for all sample countries' estimations. Once the cointegration has been detected, the study evaluates the long-run and short-run magnfititutes of explanatory variables on HCD in BRICS nations.

**Table 8:** AARDL cointegration with the interactive term.

**Empirical model**		**Test statistics**	**Brazil**	**Russia**	**India**	**China**	**South Africa**
HCD|TOR, FD, GCF, MS, FDI, TOR*GCR, TOR*FD		F*_*overall*_*	11.131****	8.816^***^	9.913^***^	9.529^***^	9.806^***^
		t_DV_	−8.112^***^	−7.673^***^	−8.313^***^	−7.812^***^	−8.406^***^
		F*_*IDV*_*	6.38^***^	8.47^***^	7.76^***^	8.91^***^	8.53^***^
*Critical value : K = 7*	1%		5%		10%		
	I(0)	I(1)	I(0)	I(1)	I(0)	I(1)	
Pesaran et al. (2001)	4.459	6.206	3.251	4.64	2.729	3.985	
Narayan (2005)	−3.96	−5.49	−3.41	−4.85	−3.13	−4.53	
Sam et al. (2019)	3.58	5.91	2.46	4.18	2.00	3.47	

***,**, * *denotes the level of insignificant at a 1%, 5% and 10%, respectively*.

The results of long-run and short-run coefficients empirical estimation with interactive term is displayed in Table 9, with Panel A for long-run coefficients, Panel B for short-run coefficients, and Panel C for residual diagnostic test. Referring to tourism effects on HCD, the study documented positive and statistically significant association in Brazil (a coefficient of 0.0741), Russia (a coefficient of 0.0911), India (a coefficient of 0. 0694), China (a coefficient of 0.158), and South Africa (a coefficient of 0.1,227). More precisely, a 10% growth in tourism development in terms of tourism receipts can result in augmenting the progress of the human capital accumulation process in BRICS nations by 0.741% in Brazil, 0.911% in Russia, 0.694% in India, 1.58% in China, and 1.227% in South Africa. Study findings suggest that continual inflows of tourism income in the economy can boost the speed of HCD and support sustainable economic growth. The short-run assessment has revealed a mixed level of association, that is, positive and negative linkage with HCD, and all the coefficients are statistically. In particular, a 10% growth in remittance receipts in the short run results in increasing the HCD by 0.648% in India and 0.21% in South Africa, whereas tourism adversely causes HCD by 0.664% in Brazil, 0.945 in Russia, and 0.513% in China.

**Table 9:** Model estimation with an interactive term for indirect effects.

	**Brazil**	**Russia**	**India**	**China**	**South Africa**
Panel-A: Long-run coefficients					
TOR	0.0741 (0.0271) [2.7341]	0.0911 (0.0196) [4.5863]	0.0694 (0.6077) [0.1142]	0.158 (0.0864) [1.8271]	0.1227 (0.1556) [0.7886]
CF	0.1248 (0.0136) [9.1286]	0.1525 (0.0776) [1.9642]	0.0579 (0.0026) [22.0228]	0.0994 (0.0756) [1.3156]	0.1317 (0.0813) [1.6199]
FD	0.1139 (0.063) [2.2209]	0.1646 (0.0691) [2.3813]	0.1357 (0.0202) [6.4451]	0.1231 (0.0184) [6.682]	0.0684 (0.0387) [1.7665]
FDI	0.0433 (0.013) [3.3262]	0.0561 (0.0185) [3.0248]	0.0167 (0.0073) [2.2637]	0.018 (0.0088) [2.043]	0.0877 (0.0502) [1.7455]
MS	−0.1409 (0.0815) [−1.7291]	0.1022 (0.0856) [1.1934]	−0.0955 (0.0399) [−2.3939]	0.0412 (0.0209) [1.9653]	−0.0414 (0.0066) [−6.1994]
TRCF	0.051 (0.0219) [2.3265]	0.0488 (0.0176) [2.7646]	0.1796 (0.0273) [6.5733]	0.0677 (0.012) [−5.613]	0.0248 (0.0039) [6.2342]
TRFD	0.0938 (0.0265) [3.5319]	0.0762 (0.0254) [−3.0003]	0.0527 (0.0157) [−3.3608]	0.0746 (0.0357) [2.0911]	0.0297 (0.8911) [0.0333]
C	−13.6698 (1.9671) [−6.9491]	9.0478 (0.3777) [23.9545]	2.0235 (0.5047) [4.0093]	−2.2622 (0.9963) [−2.2704]	3.7674 (71.5122) [0.0526]
Panel-B: Short–run coefficients					
TOR	−0.0664 (0.0395) [−1.6828]	−0.0894 (0.0296) [−3.0196]	0.0648 (0.0124) [5.1923]	−0.0513 (0.0076) [−6.7187]	0.021 (0.0031) [6.7042]
CF	−0.0243 (0.006) [−4.0584]	−0.0689 (0.0198) [−3.4802]	−0.0426 (0.0139) [−3.0573]	0.126 (0.056) [2.2485]	−0.0086 (0.0038) [−2.2331]
FD	−0.0267 (0.0093) [−2.8722]	−0.0562 (0.0175) [−3.2021]	−0.064 (0.0303) [−2.1126]	0.0425 (0.0193) [2.1973]	0.0728 (0.0125) [5.7919]
FDI	0.1905 (0.6997) [0.2722]	−0.0783 (0.0157) [−4.9594]	−0.037 (0.0081) [−4.5343]	−0.0651 (0.0221) [−2.9455]	−0.0453 (0.0184) [−2.462]
MS	0.0003 (0.001) [0.394]	−0.0848 (0.0494) [−1.7156]	0.0014 (0.0311) [0.0469]	0.046 (0.0181) [2.5305]	−0.0175 (0.003) [−5.6623]
TRCF	0.0094 (0.0009) [9.4318]	0.0687 (0.023) [2.9808]	−0.0197 (0.0106) [−1.8618]	−0.0658 (0.0174) [−3.7661]	−0.0171 (0.0239) [−0.7156]
TRFD	0.0092 (0.0024) [3.7312]	0.0135 (0.007) [1.9195]	0.0308 (0.0091) [3.3792]	−0.0544 (0.0167) [−3.2486]	0.1308 (0.023) [5.6741]
CointEq (−1)	−0.1043 (0.0213) [−4.8901]	−0.0894 (0.028) [−3.1929]	−0.1598 (0.0264) [−6.0545]	−0.1747 (0.0244) [−7.1552]	−0.459 (0.0017) [59.6272]
Panel-C: Residual diagnostic test					
	0.7995	0.6846	0.5773	0.8423	0.7953
	0.4815	0.4471	0.4281	0.6063	0.5408
	0.442	0.7738	0.5316	0.4298	0.6524
	0.8431	0.4009	0.4589	0.4431	0.471

Regarding the role of capital adequacy in human capital formation in the economy, the study has documented the positive tie with HCD in BRICS nations: Brazil (a coefficient of 0.1248), Russia (a coefficient of 0.1525), India (a coefficient of 0.0579), China (a coefficient of 0.0994), and South Africa (a coefficient of 0.1317). In particular, a 10% growth in domestic capital formation in BRICS can increase human capital accumulation by 1.248% in Brazil, 1.525% in Russia, 0.579% in India, 0.994% in China, and 1.317% in South Africa. Regarding short-run coefficients, the adverse effects run from gross capital formation to HCD in Brazil (a coefficient of −0.0243), Russia (a coefficient of −0.0689), India (a coefficient of −0.0426), and South Africa (a coefficient of −0.0086), whereas a positive and statistically significant connection was revealed in China (a coefficient of 0.126).

Referring to the association between financial development and HCD in BRICS nations, the study findings documented a positive and statistically significant tie between the measurement of financial development and HCD in the long run and mixed-effects revealed in the short run. In the long run, a 10% growth in the financial system can trigger the present state of HCD by 1.139% in Brazil, 1.646% in Russia, 1.357%, 1.231% in China, and 0.684% in South Africa. On the other hand, financial development in the short-run revealed positive and statistically significant association in China (a coefficient of 0.0425) and South Africa (a coefficient of 00728), whereas a negative and statistically significant association was found in Brazil (a coefficient of −0.0267), Russia (a coefficient −0.0562), and India (a coefficient −0.064). So, it is assumed that financial development has critical importance for sustainable HCD; thus, BRICS nations have to establish uniformity between financial policy formulation and HCD strategies in the economy.

Referring to the coefficients of interactive terms, that is, the indirect effects of tourism on HCD through the channel of financial development (TOR^*^FD) and capital formation (GCF^*^TOR), the study documented a positive and statistically significant connection between TOR^*^CF (TOR^*^FD) with HCD in the long run in Brazil with a coefficient of 0.051 (0.0938), in Russia with a coefficient of 0.0488 (0.0762), in India with a coefficient of 0.1796 (0.0527), in China with a coefficient of 0.0677 (0.0746), and in South Africa with a coefficient of 0.0248 (0.0297). For the short-run assessment, the study documented a mixed nature of association, that is, both positive and negative linkage were revealed, but the coefficients' elasticity is petty insignificant.

The speed of long-run disequilibrium correction due to short-run shocks is measured by the coefficient of the error correction term and has to be negative and statistically significant. More precisely, due to explanatory variables shock in the short run, the disequilibrium state can be rectified at a speed of 10.43% per period in Brazil, 8.94% per period in Russia, 15.98% per period in India, 17.47% per period in China, and 45.9% in South Africa. Furthermore, the study has implemented several residual diagnostic tests to confirm the model's internal consistency, robustness in estimation, and efficiency in managing residual (see panel-C). Referring to residual diagnostic test statistics, it is revealed that empirical models are free from serial correlation, and normally distributed residuals have no issue for heteroskadacity.

Next, the directional association in the empirical equation has been derived by implementing the novel TY causality test with the Fourier function familiarized by Enders and Jones (2016). The results of the causality test are displayed in Table 10. The study documented several directional causalities among the research units. However, considering the target association of tourism and HCD, the study documented feedback hypothesis that is bidirectional causality [TOR← → HCD] in all sample countries except India. The feedback hypothesis between tourism and HCD explained the complementary association between them; precisely, strategic decisions for tourism or HCD can be attributed to either side. Thus, it is suggested for the policy-making concern that, when formulating the macro policies focusing on tourism and human capital accumulation in BRICS nations, that both aspects receiving full attention needs to be ensured.

**Table 10:** Toda–Yamamoto Fourier causality test.

	**Brazil**		**Russia**		**India**		**China**		**South Africa**	
TOR ↛ HCD	8.09 [0.032]	√	16.474 [0.004]	√	5.493 [0.022]	√	14.627 [0.005]	√	10.075 [0.0002]	√
GCF ↛ HCD	14.676 [0.030]	√	13.739 [0.027]	√	12.163 [0.011]	√	1.39 [0.665]	√	8.149 [0.047]	√
FD ↛ HCD	15.258 [0.039]	√	1.744 [0.138]		0.274 [0.756]		2.31 [0.391]	√	1.013 [0.740]	
FDI ↛ HCD	0.916 [0.621]		6.924 [0.027]	√	11.786 [0.004]	√	1.826 [0.187]	√	4.078 [0.0490]	√
GCF ↛ HCD	9.512 [0.011]	√	14.233 [0.005]	√	6.632 [0.017]	√	3.624 [0.065]	√	0.637 [0.907]	
HCD ↛ TOR	7.947 [0.006]	√	4.624 [0.018]	√	0.555 [0.935]		14.651 [0.014]	√	0.031 [0.784]	√
HCD ↛ GCF	6.583 [0.044]	√	11.518 [0.009]	√	13.522 [0.008]	√	11.212 [0.005]	√	8.295 [0.0341]	√
HCD ↛ FD	0.569 [0.747]		10.887 [0.004]	√	13.55 [0.073]	√	15.083 [0.007]	√	0.148 [0.451]	√
HCD ↛ GCF	10.874 [0.002]	√	2.899 [0.278]		0.719 [0.844]		8.107 [0.037]	√	15.299 [0.064]	√
HCD ↛ FDI	12.488 [0.071]	√	8.008 [0.032]	√	7.322 [0.007]	√	13.93 [0.005]	√	2.333 [0.266]	

*The values in [ ] explain the Bootstrap p-value associated with w-statistics. √ indicates causalities*.

## Discussion

Referring to tourism effects on HCD, the study established a positive and statistically significant association between them, suggesting the booster role of tourism receipts in the process of human capital accumulation, both in the long run and the short run. The positive and statistically significant linkage was confirmed in both models without the interactive term and with the interactive term. Our findings align with existing literature such as Ngoma and Ismail (2013) and Pu et al. (2021). Tourism development has the potential to boost human capital in many ways. First, it employs individuals employed in the industry and those who provide services to foreign visitors throughout their presence (Sinclair and Stabler, 2002; Jordan et al., 2016; Folarin et al., 2017). They could afford to obtain the necessities of life *via* their job, allowing them to live a respectable existence and extending their life expectancy. Second, tourism development generates cash for the government and provides extra financing for the government to subsidize health and educational services, making them affordable to a broad segment of the population and providing educational and health facilities. Moreover, our present study contradicts the study finding documented by KoŽić (2019) that revealed that tourism development subsidized the present state of HCD by reducing school enrolment in Croatia.

The nexus between financial development and human capital accumulation established a positive tie between them, suggesting that the development in the financial system played a positive role in contributing to human capital accumulation in BRICS. Our study findings are supported by the existing literature of Hakeem and Oluitan (2012) and Arif and Khan (2019). The financial industry's growth increases the efficiency of lending and the intermediation between lenders and borrowers. Improved financial market conditions also encourage labor division and innovation, boosting efficiency, competitiveness, and, eventually, innovating. Financial development, in theory, is just as important as HCD, and both contribute considerably to the economic progress. When physical capital is paired with inadequate human capital, economic development may be slow. Physical capital contributes to human capital only when finance is permitted to “perform what finance can do,” boosting efficiency by redistributing buying power from low-return consumers to high-return users. Finance combats poverty by raising income, and over time, finance alleviates poverty through improving health and education. This is accomplished *via* investment in skill development and services and physical infrastructure that promote health and longevity. Human and physical capital drive economic development endogenously and are critical components of economic growth (Sibel et al., 2015). While both are necessary for long-run development, physical capital accumulation occurs first, and human capital buildup occurs later (Laktionova et al., 2021).

Gross capital formation revealed a positive and statistically significant association with HCD in BRICS nations, suggesting that capital adequacy induces human development in the economy. Capital accumulation is comparable or necessary to a nation's physical capital stock being increased by investment in social and economic infrastructure. Gross fixed capital accumulation comprises gross domestic private investment and gross domestic public investment and result in the production of physical items (plants, equipment, and machinery, for example) and/or intangible assets (such as a high quality and level of education, health, scientific tradition, and research) in a nation. The study of Onyinye et al. (2017) argued that capital development might result in increased output and job prospects. He also emphasized that capital creation results in technological advancement, enabling large-scale production economies to be realized, enhancing specialization, or supplying machinery, tools, and equipment for the rising labor force. Moreover, capital production aided in eliminating market defects *via* the creation of economic and social overhead capital, breaking the vicious loop of poverty on both the demand and supply sides (Shuaib and Ndidi, 2015). Bakare (2011) advocated that capital formation affects a country's economic prosperity and contributes to satisfying the needs of an expanding population in a growing economy. It results in the appropriate exploitation of natural resources and the formation of diverse enterprises, increasing levels of growth and satisfying the diverse desires of the populace, and, eventually, results in increasing the population's standard of living and economic well-being, hence boosting human capital accumulation.

## Conclusion

The motivation of the study is to gauge the role of tourism development on sustainable HCD in BRICS for the period 1984-2019. The study applied both conventional and unknown structural break unit root tests, the novel Bayer and Hanck (2013) combined cointegration test, the augmented ARDL test, offered by Sam et al. (2019), and directional causality by performing the causality test with Fourier function, familiarized by Nazlioglu et al. (2016). The key findings of the study are as follows:

First, the stationarity test with conventional and unknown structural break unit root tests revealed that all the variables were stationary either level or after the first difference but not after the second difference. Findings suggest that variables are integrated in mixed order, preferable for autoregressive distributed lagged implementation.

Second, the study implemented the novel combined cointegrated test offered by Bayer and Hanck (2013); the test statistics of all five models for BRICS are higher than the critical value offered at a 5% level of significance. Study findings suggest the long-run cointegration between tourism, financial development, gross capital formation, and HCD.

Third, empirical assessment with AARDL revealed long-run cointegration between tourism, gross capital formation, financial developer, and HCD in BRICS nations. In the long run, the coefficients of tourism, gross capital formation, and financial development are positive and statistically significant at a 1% level of significance, suggesting the contributory role toward sustainable HCD in BRICS. Furthermore, in the short run, the magnfititutes of tourism, gross capital formation, and financial development to HCD are positive and statistically significant but insignificant compared with long-run coefficients. The coefficient of joint effect, that is, the interactive terms are positive and statistically significant, implying the indirect effects of tourism through financial development and gross capital formation on HCD, especially in the long run.

Fourth, the directional causality with Fourier-TY, following Nazlioglu et al. (2016), documented the feedback hypothesis, that is, bidirectional causality running between tourism and HCD in all sample nations except in India.

Considering all the information revealed with the empirical assessment, we ended with the concluding remarks for further actions in developing and achieving sustainable HCD in BRICS nations. For maintaining sustainable HCD with the assistance of tourism, it is evident that policymakers have to put extra effort into boosting the progress of tourism development because the findings of the interactive term reveal the positive connection to HCD. HCD, according to literature, ensure a skilled workforce in the economy, that is, the transformation of the population into resources with the inclusion of technology and conceptualization development. Precisely, the population having both technical know-how and formal education, that is, population enrolment in schooling, can boost accumulating skilled human resources in the society. Furthermore, tourism inflows increase earning opportunities in the economy and support increasing present living standards. Thus, the capacity to avail education can be an alternative through which tourism can play a role in sustainable economic development in BRICS. Additionally, higher education plays a role in educational tourism, recognizing that “educational tours are an interesting site of study, first, because they are explicitly about learning, and second, because they provide an opportunity for universities to reach beyond their walls and directly teach members of the broader community.” It is asserted that universities can play a pivotal role in teaching ethics outside of the context of academic education by providing moral education that supplements professional skills and by using the entire world as a stage for pedagogy and that it is possible to increase the links between the university and the community by using mixed strategies, such as practical and experiential learning at a local level, and by exposing students to real life.

In other words, government policies for promoting tourism can increase financial activities in the financial system, suggesting that financial development and eventually financial progress allow HCD by offering opportunities, skills, and technological exposure in the economy. Financial inclusion helps tourist businesses and entrepreneurs to acquire institutional financing and bolster the resources necessary for tourism operations. Inextricably linked to innovation, access to financing contributes to economic development *via* increased productivity. Bank-based financial inclusion significantly enhances financial inclusion and contributes to faster economic development *via* inclusive growth in developing nations where bank-based financial systems are prevalent. Furthermore, financial inclusion through microfinance models lowers the cost of financial intermediation for borrowers, resulting in quicker economic growth. Tourism helps reduce poverty in developing nations, especially in the least developed countries, when people and small- and medium-sized enterprises (SMEs) active in the tourism industry have established access to financing in BRICS. Tourism development has revealed the sources of capital flows in the economy because the money flows from tourism receipts increase the capital adequacy in the economy and allow the population to earn extra by garbing the income scope available in the economy, which eventually promotes the prospects of human rights capital in the long run.

## Data Availability Statement

The original contributions presented in the study are included in the article/supplementary material, further inquiries can be directed to the corresponding author/s.

## Author Contributions

JL: introduction, methodology, and first draft preparation. MQ: introduction, methodology, empirical model estimation, and final preparation. Both authors contributed to the article and approved the submitted version.

## Funding

This research project has been funded by the Institute of Advanced Research (IAR) (Grant-IAR/2021/PUB/007).

## Conflict of Interest

The authors declare that the research was conducted in the absence of any commercial or financial relationships that could be construed as a potential conflict of interest.

## Publisher's Note

All claims expressed in this article are solely those of the authors and do not necessarily represent those of their affiliated organizations, or those of the publisher, the editors and the reviewers. Any product that may be evaluated in this article, or claim that may be made by its manufacturer, is not guaranteed or endorsed by the publisher.
